# Patient-Specific Normalized Glandular Dose Range Estimate For Mammography

**DOI:** 10.21203/rs.3.rs-8484301/v1

**Published:** 2026-01-12

**Authors:** Lacey L. Medlock, Bryce Smith, Murtuza S Taqi, Joyoni Dey

**Affiliations:** Louisiana State University; Mary Bird Perkins Cancer Center; Louisiana State University; Louisiana State University

## Abstract

Breast cancer is the most common cancer among women in the United States, and early detection is essential for reducing mortality. Screening mammography plays an important role in early detection by enabling identification of malignancies at earlier, more treatable stages. However, currently accepted dose estimation methods rely on simplified assumptions, typically modeling the breast as a homogeneous 50/50 mixture of adipose and fibroglandular tissue. Such assumptions neglect the substantial spatial heterogeneity of glandular tissue within individual breasts, most of which contain well below 50% FG tissue, and may therefore lead to inaccurate estimates of normalized glandular dose (DgN).

In this work, we first show via Monte-Carlo Simulations the range of the DgN possible by just placing the fibroglandular tissue in the top, center or bottom for a range of breast models with GF varying sizes and glandular fraction percent. Then we propose a patient-specific framework for estimating a range of normalized glandular dose (DgN) from a (single) mammographic projection and its corresponding glandular fraction (GF) map, derived by our prior method on GF estimation (Smith, Dey et al) from a single image. We demonstrate that DgN can vary by up to a factor of three depending solely on differences in depth distribution of fibroglandular tissue, even when resultant projection images are indistinguishable from one another. Using simulated projections and GF maps, a Siddon ray-tracing-based back projection algorithm was applied to generate volumetric breast reconstructions that yielded the minimum and maximum achievable DgN while preserving both the projection image and GF distribution. We analytically demonstrate that the dose to these back projected volumes can be minimized or maximized by altering the placement of fibroglandular tissue within the volume. Monte Carlo simulations including Compton scatter were then performed, and the resulting doses were normalized by entrance air kerma to obtain DgN estimates for each reconstructed configuration. The results demonstrate that realistic bounds on patient dose can be derived from limited imaging information, supporting more personalized and transparent mammographic dose assessment.

## 1. INTRODUCTION

Breast cancer is the most frequently diagnosed cancer among women in the United States and the second leading cause of cancer-related mortality in this population [1]. Because prognosis is strongly dependent on stage at diagnosis, early detection is critical. The United States Preventive Services Task Force recommends biennial screening mammography for women at average risk between 50 and 74 years of age [2]. Given that X-ray mammography is the primary imaging modality for breast cancer screening, an average-risk woman may undergo approximately 13 screening mammograms in her lifetime, contributing to more than 39 million mammographic examinations performed annually in the United States [3].

Although mammography delivers a relatively low radiation dose [4], exposure to ionizing radiation carries a small but non-zero risk of radiation-induced cancer [5]. Consequently, accurate estimation of patient dose during screening remains and important component of risk-benefit analysis in breast cancer screening.

Conventional normalized glandular dose (DgN) estimation methods rely on parameterized models that account for anode/filter combinations and assume a homogeneous breast composed of a fixed, typically 50/50 mixture of adipose and fibroglandular tissue [6]. In reality, however, glandular tissue distribution is spatially heterogeneous and varies substantially, both between patients and within individuals. Ignoring this heterogeneity can lead to systemic under- or over-estimation of dose, depending on the true distribution of fibroglandular tissue within the breast [7].

In a prior work we have shown that a pixel-wise “glandular fraction” map maybe be obtained from a single projection, based on three-tissue modeling of breast [8].

Because a single mammographic projection does not uniquely determine the three-dimensional distribution of glandular tissue, exact patient-specific dose cannot be recovered from individual standard screening images. However, we can recover an approximate range for each patient from the projection images as well as the derived glandular fraction image.

The goal of this work, then, is not to estimate a single deterministic DgN value, but rather to determine a physically feasible range of DgN values consistent with a given mammographic projection and its associated glandular fraction map. By identifying the minimum, maximum and most-likely doses under these constraints, we aim to provide clinically meaningful bounds on patient dose that better reflect anatomical uncertainty.

## 2. METHODS

The methods are divided into two parts. In the first part the issue of DgN variation due to fibroglandular fraction distribution was investigated via Monte-Carlo simulations of mammographic projections and dose for different sized breasts for 20% glandular fraction, as well as different glandular fractions (20-50%) for a single breast size. For each case the fibroglandular tissue is placed at the top, center and bottom and the normalized DgN is calculated via Monte-Carlo. In the second part it is demonstrated that starting with a singular mammographic projection image and glandular fraction, which may be obtained by our prior method [8], it is possible to estimate a range of maximum, minimum and most likely DgN by back-projection and concentrating the fibroglandular tissue at the top, bottom and center respectively. The two methods are described in the following sub sections.

### 2.1 Monte Carlo Simulation to Estimate Range of Normalized Glandular Dose (DgN)

Monte Carlo simulations were performed to quantify the range of possible DgN values arising from different depth-wise distributions of fibroglandular tissue within simulated breast objects. Projection images were generated from simulated breast objects in which fibroglandular tissue is concentrated near the top, center, or bottom of the breast.

Dose to the fibroglandular tissue as well as normalized version were calculated.

All simulations were conducted using the TOPAS (Tool for Particle Simulation) framework, a wrapper for the Geant4 Monte Carlo toolkit. The simulation pipeline consisted of spectral generation, system geometry modeling, dose scoring within fibroglandular tissue, and normalization by entrance air kerma, each explained below.

#### Spectral Generation

**Figure 1:**
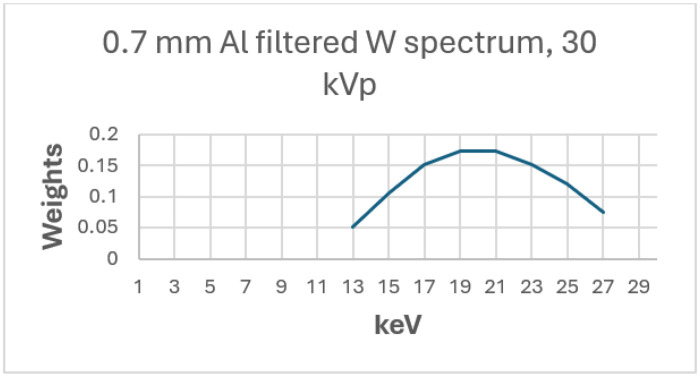
Beam weights versus keV for a tungsten anode with 0.7 mm aluminum filter operated at 30kVp. The lowest and highest energy bins are omitted because their contributions to the spectrum account for less than 5%.

To compute DgN for the various cases, a clinically representative X-ray spectrum was generated. Specifically, a 30-kVp tungsten anode spectrum with 0.7-mm aluminum filtration (W/Al) was modeled, corresponding to a common configuration used in screening mammography. The polyenergetic spectrum was discretized into 2 keV bins spanning 13-27keV (Figure 1). Energies outside this range were excluded due to their negligible contribution to dose.

#### TOPAS geometry and breast models

**Figure 2:**
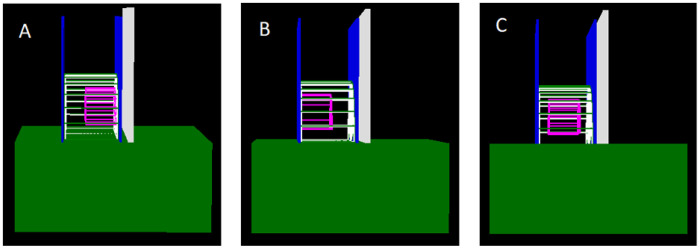
Simulated breast model with fibroglandular tissue concentrated in three locations. a) At the bottom of the breast, b) at the top of the breast, c) in the center of the breast. The green wireframe represents the skin layer. The white wireframe represents adipose tissue, and the blue wireframe represents fibroglandular tissue.

TOPAS version 3.7 with the Geant4 Option4 physics list, which includes the major photon interaction processes relevant to medical X-ray imaging–photoelectric absorption, Compton scatter, and Rayleigh scatter, was used to model a generic mammography system following established implementations in the literature [11,12]. The simulated mammography system included an X-ray source, lead beam-shaping block, water bath, Lexan compression paddles, anti-scatter grid, and a cesium iodide (CsI) detector (Figure 2).

**Figure 3:**
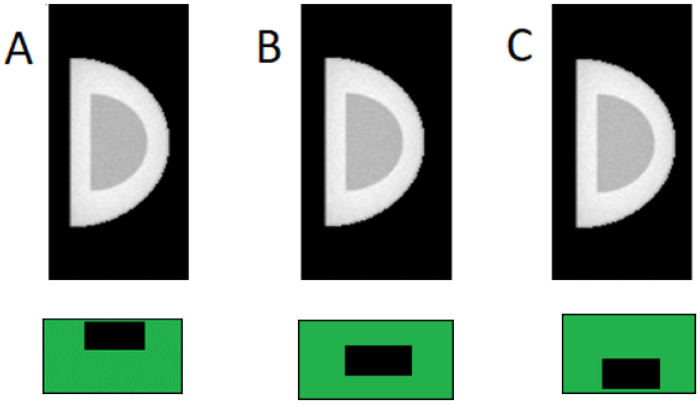
An example of projection images for a simulated breast object with fibroglandular tissue concentrated at different depths, as is illustrated by the green and black schematic at the bottom of the image. Fibroglandular tissue concentrated (a) at the top, closest to the X-ray source, (b) in the center of the simulated breast object, (c) at the bottom of the simulated breast object, farthest away from the X-ray source. Despite their visual similarity, the corresponding DgN values differ substantially, illustrating the need for a range-based dose characterization.

The simulated breast was modeled as a semi-ellipsoidal volume composed of adipose tissue, fibroglandular tissue, and a 1.45-mm skin layer. Breast diameters ranged from 11.29 to 14.29 cm, with compressed thicknesses from 3.29 to 6.29 cm (Table 1). For all breast diameters, three cases with 20% glandular fraction by volume were modeled. For the 12.29-cm diameter breast, additional simulations were performed for glandular fractions of 30% and 50% by volume. These values were selected to represent the distribution of breast compositions commonly encountered in screening mammography. For each configuration, the fibroglandular tissue was arranged in three depth distributions: concentrated near the top (source side), center, or bottom (detector side) of the breast (Figure 3).

For each 2keV bin, 120 million photons were simulated, providing sufficient signal-to-noise ratio while maintaining feasible computation times. The resultant projections were weighted by the spectrum and added to obtain resultant projections (example shown in Figure 3).

Dose to the fibroglandular tissue was recorded, and entrance air kerma was estimated by tallying photons passing through a 3 cm × 3 cm scoring plane above the upper compression paddle. The DgN is calculated as described below.

#### DgN calculation

Entrance surface air kerma was calculated for each energy according to the following equation.

(1) $$K_{E}=1.602\times 10^{-10}\times E\times 10^{-3}\times \frac{{\text{μ}}_{en}}{\rho_{\;air}}\times \frac{N}{9cm}$$

where K_E_ is entrance surface air kerma, E is energy in keV, $\frac{{\text{μ}}_{en}}{\rho_{\;air}}$ is the mass-energy absorption coefficient for dry air air at energy E, and N is the number of photons that pass through the 3 cm × 3 cm scoring plane. The air kerma for each energy was weighted by the beam weight, as was the dose for each energy. The DgN was then calculated by

(2) $$D_{g}N=\frac{\sum_{E}D_{E}W_{E}}{\sum_{E}K_{E}W_{E}}$$

where W_E_ is the beam weight for each energy and D_E_ is the dose for each energy. The sums were performed across all energy bins.

### 2.2 Reconstruction of feasible breast volumes

**Figure 4:**
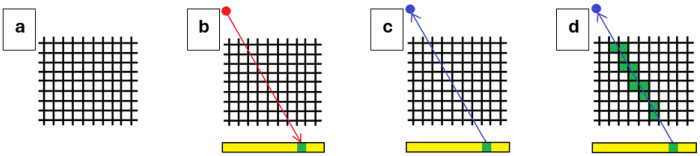
Illustration of the steps of a Siddon ray-tracing algorithm. a) An object is positioned between an X-ray source and the detector. b) The yellow bar represents the projection image, with the green box a single pixel of interest. The red arrow shows the direct photon path from the source to the detector that generates this pixel, with scatter neglected. c) The blue arrow indicates the backprojection path utilized by the ray-tracing algorithm. d) Voxels intersected by the ray corresponding to the selected projection pixel are identified and highlighted in green.

To estimate the minimum and maximum possible DgN values corresponding to a given mammography projection image, a Siddon ray-tracing algorithm [9,10] was used to back-project a ray from each detector pixel into the breast volume towards the source. Figure 4 provides a simplified, two-dimensional illustration of the reconstruction procedure. In Figure 4a the object is positioned between the X-ray source and the detector, and a projection image is generated (Figure 4b). A single detector pixel in the projection image is selected as the pixel of interest, and the corresponding ray is back-projected from the detector toward the source (Figure 4c).

All voxels intersected by this ray are identified and are subsequently assigned as adipose tissue, fibroglandular tissue, or skin, consistent with the projection as well as the glandular fraction image [8] as explained below.

To isolate the reconstruction code from possible errors in the glandular fraction estimation code [8], a “perfect” glandular fraction map was assumed, specifying the fraction of fibroglandular tissue along each ray. This map provides the total fibroglandular path length that must be satisfied for each ray, but does not specify how that fibroglandular tissue is distributed in depth. Using this information, voxels intersected by each ray were assigned as fibroglandular tissue or adipose tissue in a manner that either minimized or maximized dose, while preserving the projection image and GF map.

For each projection pixel, the ray-tracing algorithm identifies all voxels intersected by the corresponding ray. The total fibroglandular thickness associated with that ray is calculated as the product of the pixel’s GF value and the known compressed breast thickness. Initially, all intersected voxels are assigned as adipose tissue. Voxels are then iteratively assigned as fibroglandular tissue until the cumulative fibroglandular path length along the ray matches the glandular tissue thickness specified by the glandular-fraction map.

Appendix A shows an analytical proof of dose at the glandular tissue being maximized if the tissue is concentrated at the top (entrance) surface of the breast versus distributed along the depth. Minimization when the glandular tissue is at the bottom follows as a corollary.

To generate the *maximum* dose configuration, fibroglandular tissue is preferentially assigned to voxels closest to the X-ray source along each ray. Beginning at the entrance surface of the breast, voxels traversed by Siddon-ray-tracing are converted from adipose to fibroglandular tissue one at a time until the required glandular path length is achieved. The reconstruction is performed in MATLAB using 1 mm^3^ voxel size. Conversely, to generate the *minimum* dose configuration, fibroglandular tissue is assigned starting at the deepest voxels along the ray, farthest from the source, and proceeding toward the entrance surface until the same glandular path length constraint is satisfied. In both cases, this procedure assures the reconstructed volume reproduces the original projection image and GF map, while dose distributions are extremized. Similarly for the *central* configuration, adipose voxels were converted to fibroglandular starting from the central line and proceeding up and down until the glandular path length constraint is satisfied.

Once fibroglandular and adipose assignments are completed for all rays, a skin layer is added to the surface of the reconstructed breast volume. The skin is modeled as a uniform 1.45 mm thick layer across the entire surface. However, due to the 1 mm voxel resolution, the outermost voxel layer is assigned as skin, resulting in an effective skin thickness of 1 mm.

**Figure 5:**
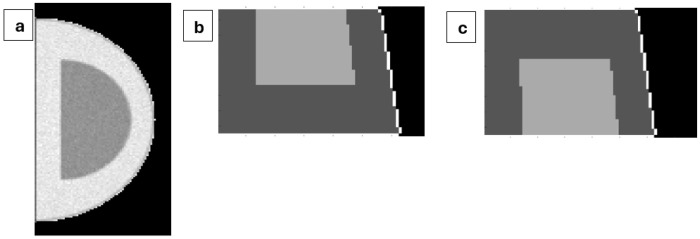
Reconstructions for a 12.29 cm × 4.29 cm breast object with a nominal glandular fraction of 20% (a) A projection image generated in TOPAS, with fibroglandular tissue concentrated at the top of the breast. (b) Cross section of the reconstructed volume optimized to maximize dose, shown in the x=95 mm plane. (c) Cross section of the reconstructed volume optimized to minimize dose, shown in the same plane.

Cross sections of representative reconstructed breast volumes are shown in Figure 5. Figure 5a shows the projection image generated in TOPAS for a 12.29 × 4.29 cm breast with a nominal glandular fraction of 20%. Figures 5b and 5c show cross sections through the reconstructed volumes optimized to maximize and minimize dose, respectively. In both cases, the cross section corresponds to the x = 95 mm plane, which bisects the reconstructed object.

In both reconstructed volumes, the proximal (left) boundary of the reconstructed breast object is parallel to the z-axis, reflecting the chest wall. However, the distal (right) boundary is slanted, which is expected from the divergent ray-tracing geometry used in the back-projection. Since the source is located 35.46 cm above the top surface of the breast at the chest wall, rays connecting detector pixels to the source are angled relative to the z-axis, resulting in a sloped distal surface in the reconstructed cross sections.

## 3. RESULTS

### 3.1 Estimation of DgN

**Figure 6:**
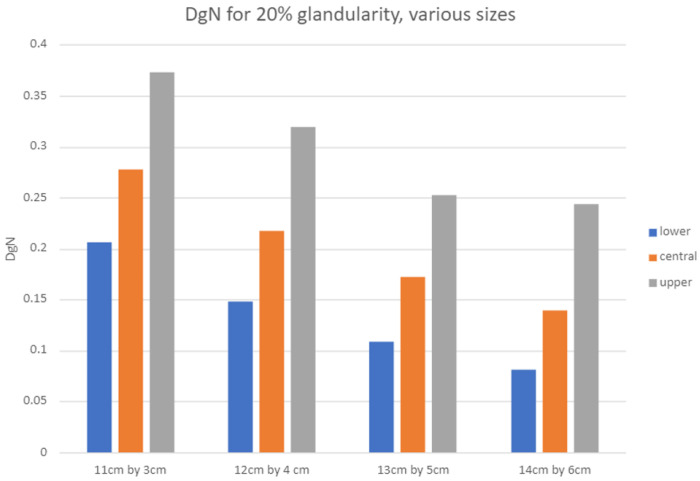
DgN values for 20% glandularity for breasts of various dimensions. Note that with increased breast thickness, the ratio between maximum and minimum DgN increases, with the ratio rising from 1.8 for a 3.29 cm thick breast to nearly 3.0 for a 6 cm thick breast

Figure 6 summarizes DgN values for breasts of varying thickness at 20% glandularity. For all fibroglandular distributions, the DgN decreases as the thickness of tissue increases due to increased attenuation and greater average depth of fibroglandular tissue. However, the ratio between maximum and minimum DgN increases with thickness, from approximately 1.8 for a 3.29 cm thick breast to nearly 3.0 for a 6 cm thick breast.

**Figure 7:**
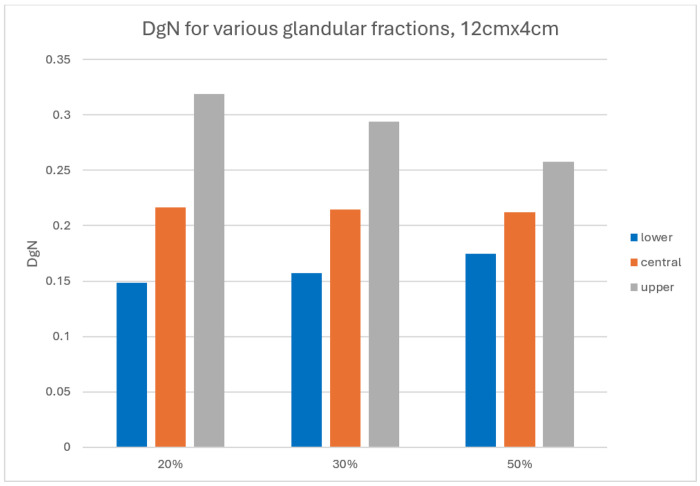
DgN values for varying glandular fractions, breast dimension including skin 12.29cm x 4.29cm. The difference between maximum and minimum is reduced as the glandular fraction is increased and occupies a larger fraction of the breast volume.

Figure 7 shows DgN values as a function of glandular fraction for a fixed 4.29 cm thick breast. While the DgN remains approximately constant despite increasing glandularity, for the case where the fibroglandular tissue is concentrated at the center of the breast, the range between minimum and maximum DgN narrows. This is expectedly a reduced sensitivity to fibroglandular depth distribution when glandular tissue occupies a larger fraction of total breast volume.

### 3.2 Reconstruction from Projection and GF map

After reconstructing the breast object using ray tracing to place the fibroglandular tissue in the top (maximize DgN), bottom (minimize DgN) and center (likely DgN), consistent with the glandular fraction map, the objects are simulated in TOPAS to ascertain the values of DgN after reconstruction and to compare with our original values. The TOPAS geometry is maintained the same as Fig. 2, with the breast object inserted as the voxelized reconstructed phantom (composed of thousands of 1 mm^3^ voxels, each assigned as adipose tissue, fibroglandular tissue, skin, or air, as determined by the ray-tracing algorithm).

**Figure 8:**
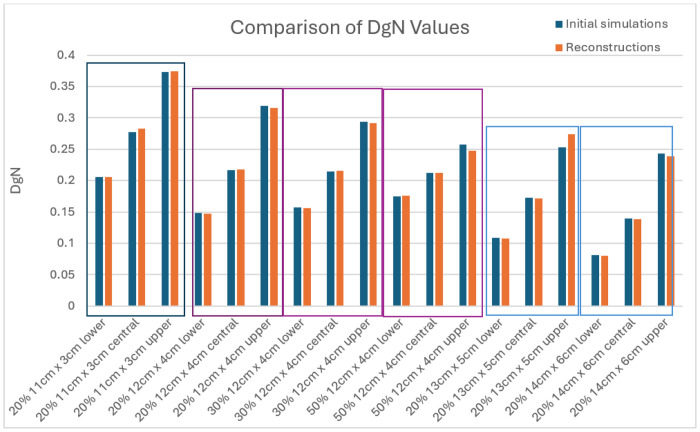
Comparing DgN values from the initial (original) simulations and the corresponding reconstruction. Groups of three (lower, central and upper) are grouped with rectangular boxes (also color-coded for size) for readability.

The original simulated breast object DgN values are compared against reconstructed simulated breast object DgN values. The numerical DgN results are tabulated in Appendix A2 for 4 different sizes. The bolded results (where original and reconstruction are in the same configuration, that is either lower, central or upper) are shown in Fig. 8, for comparison. For all 18 baseline cases, the true DgN from the original breast configuration (centrally located) lay within the reconstructed bounds. The largest deviation between the expected DgN and the reconstructed DgN occurs for the 13 cm × 5 cm case when the fibroglandular tissue is concentrated at the top of the breast near the source; in this instance, the discrepancy is about 8%.

## 4. DISCUSSION

This work demonstrates that substantial uncertainty (even as much as three times variation) in normalized glandular dose can arise solely from unknown depth-wise distribution of fibroglandular tissue, even when the breast thickness, glandularity, and projection appearance remain fixed. The observed variation in DgN, particularly for thicker breasts, highlights the limitations of conventional homogeneous-50/50-breast dose models and emphasizes the importance of patient-specific dose characterization.

The proposed reconstruction-based framework was verified to provide accurate DgN values (maximum deviation 8%) and provides a practical method for estimating realistic bounds on DgN from a single mammographic projection image. Rather than attempting to assume a three-dimensional geometry, the method acknowledges inherent uncertainty in mammography and quantifies its dosimetry implications. This range-based approach is particularly relevant for thicker and less glandular breasts, where dose variability is greatest.

A few limitations should be noted. First, idealized glandular fraction maps were used to decouple reconstruction performance from glandular fraction estimation error. In clinical practice, uncertainties in GF estimation will widen dose bounds. Second, voxelization and simplified skin modeling introduce small discrepancies between reconstructed and reference projections. Future work will focus on extending the framework to multi-dimensional acquisitions.

## 5. CONCLUSION

We have presented a patient-specific method for estimating feasible ranges of normalized glandular dose in mammography using a single projection image and its associated glandular fraction map. Monte Carlo simulations demonstrate that DgN may have high variation due solely to fibroglandular tissue depth distribution. The proposed reconstruction-based approach provides a practical framework for estimating anatomically-informed bounds on DgN from a single mammographic projection and represents a step toward more personalized and accurate dosimetry in breast cancer screening.

## Supplementary Material

This is a list of supplementary files associated with this preprint. Click to download.

• APPENDIXA.docx

## Figures and Tables

**Table 1.** Dimensions of the simulated breast for Monte Carlo modeling in TOPAS

Diameter (including skin)	Thickness (including skin)	Chest wall to nipple distance (including skin)
11.29 cm	3.29 cm	5.645 cm
12.29 cm	4.29 cm	6.145 cm
13.29 cm	5.29 cm	6.645 cm
14.29 cm	6.29 cm	7.

## Data Availability

The data generated and analyzed during this study are available from the corresponding author upon reasonable request.
